# Sepsis-Induced myocardial dysfunction: heterogeneity of functional effects and clinical significance

**DOI:** 10.3389/fcvm.2023.1200441

**Published:** 2023-07-14

**Authors:** Tatyana Shvilkina, Nathan Shapiro

**Affiliations:** Beth Israel Deaconess Medical Center, Harvard Medical School, Boston, MA, United States

**Keywords:** sepsis, myocardial dysfunction, septic cardiac dysfunction, echocardiography, septic cardiomyopathy

## Abstract

Sepsis is a life-threatening disease state characterized by organ dysfunction and a dysregulated response to infection. The heart is one of the many organs affected by sepsis, in an entity termed sepsis-induced cardiomyopathy. This was initially used to describe a reversible depression in ejection fraction with ventricular dilation but advances in echocardiography and introduction of new techniques such as speckle tracking have led to descriptions of other common abnormalities in cardiac function associated with sepsis. This includes not only depression of systolic function, but also supranormal ejection fraction, diastolic dysfunction, and right ventricular dysfunction. These reports have led to inconsistent definitions of sepsis-induced cardiomyopathy. Just as there is heterogeneity among patients with sepsis, there is heterogeneity in the cardiac response; thus resuscitating these patients with a single approach is likely suboptimal. Many factors affect the heart in sepsis including inflammatory mediators, catecholamine responsiveness, and pathogen related toxins. This review will discuss different functional effects characterized by echocardiographic changes in sepsis and their prognostic and management implications.

## 1. Introduction

**Figure 1:**
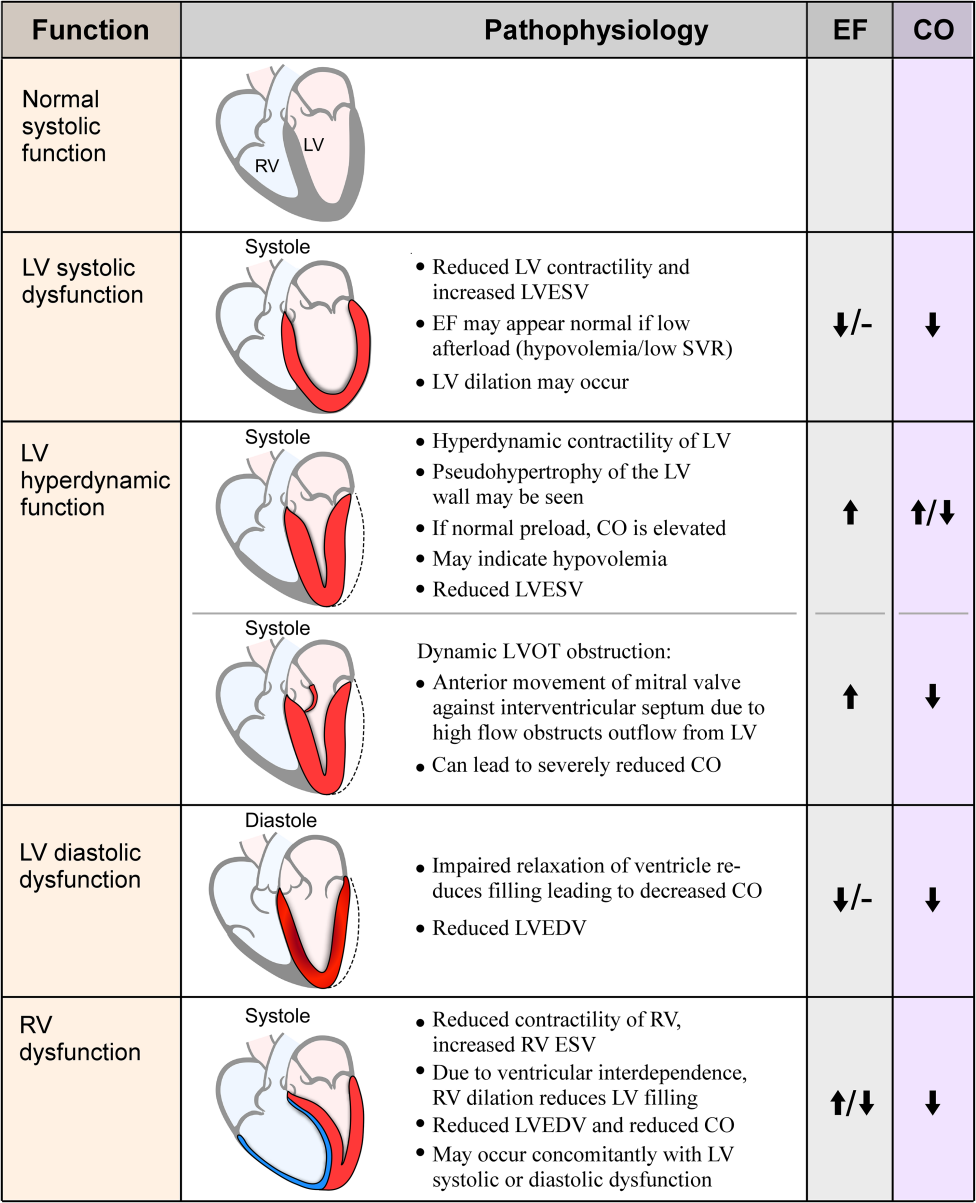
Visual representation and pathophysiology of echocardiographic findings in sepsis. EF, ejection fraction; CO, cardiac output; RV, right ventricle; LV, left ventricle; LVESV, left ventricular end-systolic volume; SVR, systemic vascular resistance; LVOT, left ventricular outflow tract; LVEDV, left ventricular end-diastolic volume; RVESV, right ventricular end-systolic volume.

Sepsis is a life-threatening disease state characterized by organ dysfunction and a dysregulated response during infection, with a mortality rate of approximately 27% worldwide (1). The heart is one of many organs affected in sepsis, in an entity called sepsis-induced cardiomyopathy, or sepsis-induced myocardial dysfunction, which occurs in 10%–70% of patients with sepsis (2). This range in incidence is wide likely due to differing definitions and echocardiographic techniques used to assess function, timing of echocardiography assessment during the clinical course, and the potential dynamic nature of cardiac functional changes. Fluid resuscitation, inotrope and vasopressor therapy, and mechanical ventilation can alter venous return, cardiac contractility, pulmonary vascular resistance, and left ventricular afterload, which affect traditionally measured echocardiographic parameters. In the 1980s Parker and colleagues demonstrated reversible depression in left ventricular ejection fraction (LVEF) and ventricular dilation in a subset of patients with sepsis (3). These characteristics are used most frequently to describe sepsis-induced myocardial dysfunction (3, 4). Subsequently, with advances in echocardiography, this term has expanded and other cardiac abnormalities are described and included such as diastolic dysfunction, right ventricular (RV) failure, and supranormal LVEF (Figure 1). Although no formal definition exists, sepsis-induced myocardial dysfunction is generally characterized as an acute process that is not caused by acute coronary syndrome and is typically reversible in seven to ten days which manifests as biventricular systolic and/or diastolic dysfunction with left ventricular dilation that is poorly responsive to fluid and catecholamines (5).

There are many events that occur during sepsis affecting cardiac performance and with advances in echocardiography we see considerable heterogeneity in this group. There is potential benefit to a more individualized approach to the hemodynamic resuscitation of this diverse patient group. In addition to reviewing echocardiographic features observed in patients with sepsis, this review will discuss their implications for treatment and prognostication as well as possible mechanisms for the various cardiac responses. Patient and disease specific factors may result in the differences seen in the cardiac responses to sepsis, and an understanding of these should help drive a more individualized approach to care.

## 2. Echocardiographic features in patients with sepsis

### 2.1. Depressed left ventricular systolic function

While the typical clinical picture of the heart in sepsis is a hyperdynamic state with elevated cardiac output (CO), since the 1950s it was recognized that a low CO syndrome exists in some patients (6, 7). Studies have outlined numerous mechanisms to explain the reduction of CO and LV systolic function: inflammatory factors (e.g., Toll-like receptors and interleukins), mitochondrial damage, abnormal calcium handling, catecholamine desensitization, and aberration of coronary microvascular blood flow due to endothelium abnormalities, neutrophil aggregation, and procoagulant factors (8–17).

In the 1980's Parker and colleagues published findings introducing the concept of LV dilation and LVEF depression in a subset of patients with sepsis that reversed to normal within two weeks as patients recovered (3, 4). In those studies, patients with ventricular dilation and lower LVEF had lower mortality compared to patients who did not have these features. This finding was also observed in animal studies (18). The theory behind this finding is that CO is maintained in the setting of low EF by dilation of the ventricles to maintain stroke volume (SV), provided that volume resuscitation is sufficient (3). Other authors also observed reduced LV end-diastolic volume (LVEDV) in non-survivors despite adequate fluid loading; however, these authors did not observe LV dilation comparable to the Parker studies in survivors (19). The concept of ventricular dilation was subsequently challenged, as the restrictive nature of the pericardium should limit significant ventricular dilation (20). Subsequent studies failed to reproduce these findings (19, 20). This discrepancy may be due to differences in techniques used to measure LVEF and CO, however it remains unclear whether a lower LVEF is cardioprotective or represents a deleterious state (19, 20).

Most recent studies use echocardiography to evaluate LV systolic function, using <40%–50% as a definition for decreased LVEF. Studies have yielded mixed results with respect to outcomes. Some studies found that decreased LVEF was associated with increased mortality (21), others reported improved survival (19), and others found no difference in survival based on LVEF (22–24). A 2013 meta-analysis showed no difference in survival based on presence of low LVEF or LV dilation (25).

A challenge with using LVEF is its dependence on preload and afterload conditions. A patient with significant contractile dysfunction can appear to have a normal LVEF in a setting of hypovolemia or low mean arterial pressure, as is often the case in sepsis. LVEF can change rapidly and significantly depending on arterial pressure and volume status, without reflecting the change in true contractility of the myocardium (26). Thus, it is not surprising that studies based on LVEF have shown such variability in prognostication.

The more recently developed speckle-tracking echocardiography (SPE) is less dependent on loading conditions and is potentially more reflective of intrinsic myocardial function (27). SPE shows myocardium movement by tracking ultrasound echoes in the myocardium throughout the cardiac cycle. Strain is the difference between the length at rest and the final length, and represents contractility (28). There are differences in global longitudinal strain (GLS) values depending on the vendor and software so at this time there is no uniform consensus, but normal is around −20% per the American Society of Echocardiography, with less negative numbers indicating decreased contractility (29). Studies on GLS as a predictor of outcome have shown more uniform results than LVEF, with a recent meta-analysis showing that lower strain (less negative values) i.e., lower contractility, is associated with higher mortality (28, 30). In this same study LVEF was not associated with mortality differences. GLS is more sensitive than LVEF, and declining cardiac contractility by GLS is observed before LVEF or decreased CO is apparent (28). With earlier identification of dysfunction, even at a subclinical level, the question remains of how to interpret and integrate this into management decisions, and further research is needed on the significance of these findings.

There is little evidence to guide management of patients with depressed systolic function. It is an unresolved question whether the dysfunction is deleterious or protective, and accordingly how aggressively clinicians should intervene to improve systolic function in the acute setting, as opposed to treating the underlying sepsis and allowing function to recover over time. In patients with sepsis-induced myocardial dysfunction and ongoing hypoperfusion after adequate volume resuscitation the Surviving Sepsis Campaign guidelines suggest inotropic therapy, with either addition of dobutamine to norepinephrine, or epinephrine alone, based on low quality of evidence (31). Dobutamine therapy is associated with improved cardiac function parameters (32), but numerous studies including network meta-analyses show varying results with respect to mortality benefit (32–37). Use of dobutamine to achieve supranormal cardiac index has shown harm (38). Prior studies were performed in a heterogeneous septic shock population, without classification or selection by echocardiography, so it is possible there is a subgroup of patients with decreased cardiac contractility that would benefit from inotropic medications. There is an ongoing trial of dobutamine for treatment of patients with septic shock and septic cardiomyopathy with reduced LVEF which may provide clarity (NCT04166331).

### 2.2. Hyperdynamic left ventricular systolic function

The hyperdynamic heart with low systemic vascular resistance is considered the typical cardiac response to sepsis. Hyperdynamic cardiac function can be a compensation for vasoplegia or insufficient intravascular volume, but can also be seen in a hyperadrenergic state from excess endogenous or exogenous catecholamine stimulation.

Investigations of hyperdynamic LV function show increased mortality (20, 39, 40). When comparing groups with low, normal, and supranormal LVEF, Chotalia and colleagues found in a study of just over one thousand patients that those with supranormal (greater than 70%) LVEF had worse outcomes compared to those with normal or even low LVEF (39). In this study a supranormal EF was associated with lower SVR so it is possible this finding represented a group with persistent vasoplegia which was potentially the driver of mortality. These patients also had higher heart rates, so the hyperdynamic state may have resulted from excessive catecholamine release. It is a plausible hypothesis that this subset of hyperdynamic patients was the reason that those with lower EF seemed to fare better in comparison in earlier studies.

While tachycardia does not necessarily indicate a hyperdynamic LVEF, there is an association seen (39). Beta-blockers have been studied to reduce heart rate and increase filling time to improve SV in patients with inappropriate tachycardia. In a meta-analysis of 7 studies including 613 patients, the use of short-acting beta-blockers esmolol or landiolol in septic patients with tachycardia after resuscitation resulted in a decreased mortality (41). While these studies do not specifically investigate patients with hyperdynamic LVEF, beta blockers may represent a potential therapy for hyperdynamic LVEF but further research is warranted. There is concern that beta blockade can suppress compensatory tachycardia in the setting of low preload, causing a drop in CO and worsening hemodynamics. A potential approach to optimize risks and benefits is to identify which patients are persistently tachycardic due to hyperadrenergic states vs. due to inadequate preload, and administer targeted beta-blocker therapy only to the hyperadrenergic population. One such suggested method is using strain echocardiography, as patients with tachycardia in the setting of high preload were observed to have worse LV strain values, which identifies a subpopulation with tachycardia due to hyperadrenergic state (42).

A specific finding in some patients with hyperdynamic cardiac function is dynamic left ventricular outflow tract obstruction (LVOTO), where blood rapidly flowing through an underfilled ventricle causes anterior motion of the mitral valve and obstruction of flow through the outflow tract, similar to the obstruction seen in hypertrophic obstructive cardiomyopathy (Figure 1). LVOTO is observed in 22%–30% of septic shock patients and can occur due to high catecholamine states, inotrope administration, and hypovolemia, and typically indicates fluid responsiveness; studies show an association between LVOTO and mortality (43, 44). There is an important clinical implication as catecholamine administration typically worsens the obstruction, resulting in unintended worsening of hemodynamic status. Authors have suggested using vasopressin instead of adrenergic agents in these patients, with one small study finding hemodynamic and respiratory improvement using vasopressin and down-titrating norepinephrine in patients with severe LVOTO (45). Beta-blockers are also a plausible treatment for this patient group, as they are recommended for treatment of LVOTO in other conditions (46, 47). There are few data on their use in the septic population specifically but are a promising therapy (48–50). None-the-less, the optimal approach is in need of further research.

### 2.3. Left ventricular diastolic dysfunction

Sepsis can lead to abnormal ventricular relaxation due to elevated catecholamines, tachycardia, and abnormalities of calcium uptake in the sarcoplasmic reticulum which impairs the active relaxation process in cardiac myocytes (51). In the healthy heart, frequency-dependent acceleration of relaxation is observed during tachycardia to maintain ventricular filling. In sepsis this can become deranged due to disruption of normal calcium transients, and result in inadequate filling (51). Excessive or rapid fluid loading can also cause impaired diastolic function and elevated filling pressures (52). Diminished relaxation of the ventricle leads to reduced filling time, decreasing SV and coronary perfusion. Diastolic dysfunction is seen in almost half of patients with sepsis (53).

Diastolic dysfunction in sepsis is commonly assessed measuring doppler flow through the mitral valve at early diastole (E) and late diastole (A), the ratio of E/A, or by using tissue doppler imaging (TDI) to measure the velocity of the myocardium at the lateral or septal aspect of the mitral valve annulus (e'). The ratio of E/e' is used as a measurement of filling pressure (54). Lateral e' <10 cm/s and septal e'< 7 cm/s indicate dysfunction (55). The ratio of E/e' ≥13–15 is used as a measurement of elevated filling pressure (54). Although there is concern for loading conditions affecting parameters of diastolic function, TDI, particularly the lateral e' measurement, seems to be relatively unaffected by preload alterations (56). A meta-analysis of 16 studies shows an association between higher mortality and lower e' values and higher E/e' ratios which are both indicative of diastolic dysfunction (23, 53, 57).

Patients with diastolic dysfunction are particularly prone to worsening hemodynamics in the setting of tachycardia and reduced filling time (58). This group may benefit from heart rate reduction in the setting of inappropriate tachycardia but as mentioned previously more research is needed to identify these patients. Reduced compliance of the LV may make fluid loading potentially harmful in this group. If filling pressures are elevated and LV cannot relax and fill adequately in diastole to augment SV, pressure increases can cause pulmonary vascular congestion (53). However, diastolic dysfunction does not necessarily indicate fluid nonresponsiveness. In fact in one study, patients with diastolic dysfunction who were fluid responsive had improvement of LV relaxation as evidenced by an increase in the early diastolic mitral annular velocity E', compared to those who were not fluid responsive, when challenged with volume expansion (59). Since relaxation is an energy dependent process, this improvement may be from enhanced coronary artery perfusion from an increased SV (59). Thus, patients with diastolic dysfunction may particularly benefit from assessment of fluid responsiveness.

### 2.4. Right ventricular dysfunction

Many factors affect the RV during sepsis, in addition to those that affect the LV as described earlier. Pulmonary vasoconstriction can occur with hypoxia, hypercarbia, and acidosis which are common in sepsis (60–62). Systemic hypotension can be further detrimental and cause pulmonary vasoconstriction due to adrenergic stimulation (60). Sepsis contributes to pulmonary vasoconstriction due to elevation of factors such as endotoxin, endothelin, thromboxane, and IL-6 (63). Mechanical ventilation, increased positive end expiratory pressure, and acute respiratory distress syndrome (ARDS) also increase RV afterload. Since the RV is used to a low pressure, high compliance circuit of the normal pulmonary vasculature, it is not well equipped to handle large increases in afterload, so these factors can contribute to RV dysfunction.

Echocardiographic assessment of RV function in sepsis is conventionally defined as tricuspid annular plane systolic excursion (TAPSE) <1.6cm, TDI S'<10cm/s, increased RV/LV size ratio, and fractional area change (FAC) <35% (64). Using strain echocardiography, RV function can be assessed globally with GLS, or by RV free wall strain which does not include interventricular septal function which is affected by the LV. Although studies suggest increased prognostic value of strain imaging for the RV, it is not yet in widespread use and the choice of imaging remains unresolved (65–68). RV dysfunction is observed in approximately one third to one half of patients with sepsis and septic shock, even up to 72% using STE (67–70). Higher mortality is observed in septic patients with RV dysfunction, with a study of 393 ICU patients showing 31% 28-day mortality compared to 16% in those without RV dysfunction (67) Consistent with this, in a study of 252 Emergency Department patients with sepsis, Innocenti et al. observed a 44% 28-day mortality in patients with RV dysfunction compared to 23% in those without (70).

RV dysfunction has therapeutic implications as well. Although fluids are recommended in sepsis to maintain adequate preload, clinicians should administer fluids with caution as a dilated RV with poor systolic function and high afterload will often have difficulty accommodating an increase in volume. RV dysfunction is a demonstrated predictor of a lower probability of fluid responsiveness, so high volume fluid resuscitation is likely less helpful in these patients which supports prioritization of vasopressors in this population; albeit, there is a lack of definitive clinical data supporting this hypothesis (71).

Although vasoactive agents such as epinephrine and norepinephrine have been shown to increase pulmonary vascular resistance in in-vitro studies (72, 73) their effects *in vivo* are less clear. Norepinephrine is the first line vasopressor recommended in sepsis. Norepinephrine increases RV systolic function without a detrimental effect on pulmonary vascular resistance which is a favorable profile for use in patients with RV dysfunction (31, 74, 75). Vasopressin has a neutral or even vasodilatory response on the pulmonary vasculature and may be a good option as well in patients with RV dysfunction, but more data are needed (76–78). There is a paucity of evidence for how to treat RV dysfunction in sepsis and this represents an important opportunity for research.

## 3. Future directions: reducing heterogeneity in sepsis

Recent efforts have focused on defining subgroups of patients within the sepsis population with the goal of identifying targeted therapeutic approaches. Several groups have attempted to define subgroups using clinical data such as demographics, organ dysfunction, vital signs, and laboratory values (79–82). Others have investigated gene expression, showing differential expression of genes related to innate and adaptive immune cell function, endotoxin tolerance, and metabolic pathways (83–86). Similar approaches are implemented in other pathologies such as ARDS, with identification of a hyperinflammatory and a hypoinflammatory subphenotype, yielding important information about response to management strategies based on subphenotype (87, 88).

A recent cluster-based study of patients with sepsis by Geri and colleagues aimed to identify cardiovascular phenotypes, using a combination of echocardiographic and clinical data to determine five cardiovascular clusters (89). In this study of 360 patients, the groups were: (a) “well resuscitated” group: no dysfunction and not fluid responsive, (b) LV systolic dysfunction group: low LVEF and cardiac index, high lactate levels and norepinephrine doses, and fluid nonresponsive, (c) hyperkinetic group: elevated LVEF and not fluid responsive, (d) RV failure group: normal or high LVEF, fluid unresponsive and associated with lower PaO2/FiO2 ratios, and (e) hypovolemic group: increased LVEF and fluid responsive despite receipt of the largest amount of fluids in this group (89). Understanding differences between cardiac responses to sepsis and how to best treat them may help inform a more individualized hemodynamic resuscitation. For instance, limiting fluids in patients with LV systolic dysfunction and RV dysfunction, using inotropes only for those patients with systolic dysfunction, prioritizing vasopressin in RV dysfunction, and using beta blockers and ensuring adequate fluid resuscitation for hyperdynamic function and LVOTO, are all theoretical ways to optimize cardiac function in sepsis, but more data are needed.

## 4. Conclusion

In summary, there are several cardiac abnormalities in sepsis identified by echocardiography. Questions remain regarding the best diagnostic and management strategies of sepsis-induced myocardial dysfunction. Are our current definitions the best way to define sepsis-induced myocardial dysfunction? Can identification of cardiac subphenotypes to guide resuscitation result in better outcomes? A better understanding of individual patient responses in the sepsis state is an exciting potential for individualized and targeted care. This, combined with evolving technological echocardiographic assessment of the heart, holds potential for future transformative personalized approaches to resuscitation in sepsis.
